# A Comprehensive Review of Electrolyte Imbalances and Their Applied Aspects in Dermatology

**DOI:** 10.7759/cureus.81353

**Published:** 2025-03-28

**Authors:** KavyaDeepu R.M., Mohnish Sekar

**Affiliations:** 1 Dermatology, Chettinad Hospital and Research Institute, Chennai, IND; 2 Dermatology, Venereology, and Leprosy, Karpaga Vinayaga Institute of Medical Sciences and Research Center, Chengalpattu, IND

**Keywords:** calcium, dermatology, electrolyte imbalance, magnesium, phosphate, potassium, sodium

## Abstract

Electrolytes play a pivotal role in the maintenance of neutrality in the minerals of the cells - also, the generation and conduction of action potentials in neurons and muscles. Key electrolytes include sodium, potassium, chloride, magnesium, calcium, and phosphate. Electrolyte imbalances can result in elevated or diminished levels. Abnormal electrolyte levels, whether raised or decreased, interfere with normal physiological activities and may result in life-threatening consequences. Electrolyte imbalances are common in critical care units, although they are less frequent in dermatological conditions. Dermatologists should, however, be knowledgeable about the skin disorders and medications that are related to or may increase the risk of electrolyte imbalance to ensure that appropriate treatments are implemented immediately to avoid negative results. The objective of this review is to narrate the dermatological features of disorders involving abnormalities of sodium, potassium, calcium, phosphorus, and magnesium.

## Introduction and background

Electrolytes are charged molecules crucial for regular metabolic processes and maintaining homeostasis. They control the action of hormones, bone structure, neuronal conductivity, muscular contraction, acid-base and fluid balance, and cell membrane function. An electrolyte level significantly above or below the expected range results from various pathophysiological factors, including acute illnesses, medications, concurrent acid-base imbalances, nutritional status, and other comorbid conditions, particularly renal disease [1,2]. Electrolyte imbalances are prevalent in critical care units, whereas they occur less frequently in dermatological settings. Dermatologists should, however, be cautious about the dermatological conditions and medications associated with or potentially increasing the risk of electrolyte imbalances to ensure that appropriate measures are implemented promptly to prevent adverse outcomes. This review consolidates the dermatological aspects of abnormalities associated with sodium, potassium, calcium, phosphorus, and magnesium.

## Review

Sodium

The serum sodium ion is the predominant cation in extracellular fluid (ECF), with a typical range of 135-145 mmol/L. It is essential for maintaining ECF volume, regulating acid-base balance, stabilizing osmotic pressure, preserving cell membrane potential, and modulating various physiological processes [3].

Hyponatremia

Hyponatremia is defined by a serum sodium concentration that falls below 135 mEq/L. Hyponatremia correlates with negative outcomes, such as prolonged hospitalization and increased mortality rates [4]. Hyponatremia may present with a range of symptoms, from mild and nonspecific manifestations like weakness, nausea, headaches, vomiting, and somnolence to severe and potentially life-threatening conditions, including intracranial hypertension, seizures, and cardiorespiratory distress [5,6]. Hyponatremia can be hypovolemic, euvolemic, or hypervolemic. Hypovolemic hyponatremia, characterized by a reduction in total body water accompanied by a more significant decrease in sodium levels, can result from renal causes such as diuretic therapy or mineralocorticoid deficiency or from non-renal losses, including vomiting, diarrhea, third spacing, or bowel obstruction. Euvolemic hyponatremia, characterized by increased total body water with normal sodium levels, is encountered with conditions such as syndrome of inappropriate antidiuretic hormone (ADH) secretion (SIADH), hypothyroidism, adrenal insufficiency, stress, and certain medications. In contrast, hypervolemic hyponatremia, defined by increased total body water relative to sodium levels, is observed in heart failure, cirrhosis, hypoalbuminemia, and renal failure [7]. Dermatological conditions and drugs associated with hyponatremia are discussed next.

Acute Skin Failure

Dermatological emergencies, including erythroderma, Steven Johnson syndrome (SJS), toxic epidermal necrolysis (TEN), acute generalized exanthematous pustulosis (AGEP), infections such as Staphylococcal skin scalded syndrome, and immunobullous disorders like Pemphigus vulgaris and foliaceus, can lead to complete dysfunction of the skin, referred to as acute skin failure [8]. Percutaneous water loss in patients with acute skin failure significantly exceeds the average total water loss of 400 mL/day. This results from compromised skin barrier function and increased fluid loss through the skin, which correlates with the elevated basal metabolic rate [9]. Adult patients with TEN exhibiting approximately 50% body surface area (BSA) involvement typically encounter a daily fluid loss of 3-4 L [10]. Inadequate fluid replacement results in a decrease in intravascular volume and the production of hyperosmolar urine. The outcomes consist of dehydration, diminished urinary output, hypovolemic hyponatremia, and several electrolyte imbalances, notably hyperkalemia and increased blood urea and creatinine levels, which are indicative of prerenal uremia. Enhanced sodium, potassium, and chloride loss is observed in blister fluid from individuals suffering from autoimmune bullous illnesses and TEN [11]. Replenishment of lost intravascular fluid and gradually restoring total body water and electrolytes are the standard protocols for treating shock. Colloids (such as fresh frozen plasma or human albumin) and normal saline (NS) are the preferred initial fluids [8].

Cutaneous Malignancies

Cutaneous malignancies, including Merkel cell carcinoma (MCC), are associated with paraneoplastic phenomena such as SIADH. This occurs even in the absence of osmotic or non-osmotic stimuli, leading to euvolemic hyponatremia, where total sodium levels remain normal while total body water increases due to pathological, non-osmotic vasopressin release. Some suggest that hyponatremia in MCC is coincidental rather than paraneoplastic [12]. Fluid restriction, tumor removal, and postoperative chemotherapy or radiotherapy have successfully resolved symptoms [13]. In a case report by Anzai et al. [14], the MCC and peripheral blood both had elevated adrenocorticotropic hormone (ACTH) levels, but the ACTH levels of the tumor were significantly lower than those of other ectopic ACTH-producing tumors, suggesting that the patient’s postoperative hyponatremia was caused by surgical stress and indapamide. Other dermatological and nondermatological malignancies presenting with dermatomyositis as paraneoplastic syndrome are listed in Table 1.

**Table 1:** Studies reporting SIADH-induced hyponatremia alongside dermatomyositis as a paraneoplastic phenomenon in malignancies. PNS, paraneoplastic syndrome; SIADH, syndrome of inappropriate antidiuretic hormone secretion; SCC, squamous cell carcinoma

Study	PNS	Seen in
Jones et al. [15]	SIADH presenting as hyponatremia and dermatomyositis	Cervical cancer
Yucel et al. [16]	SIADH presenting as hyponatremia	Cervical SCC
William [17]	SIADH presenting as hyponatremia and Dermatomyositis	Carcinoma of bronchus

Rarely, dermatomyositis can cause hyponatremia without being a paraneoplastic phenomenon. Grinnell et al. [18] reported dermatomyositis with anasarca and hyponatremia [18]. SIADH, along with hyponatremia and Leser-Trélat syndrome, represents rare paraneoplastic manifestations of renal malignancies. Causes of hyponatremia are attributed to malignancy, independent of the SIADH, adrenal failure, and cerebral or renal salt wasting [19].

Porphyria

Hyponatremia associated with SIADH occurs in approximately 90% of patients experiencing an acute porphyria attack [20]. Aminolevulinic acid (ALA) and porphobilinogen (PBG) induce abdominal pain through vascular spasm, directly stimulating ADH production from the parvocellular region of the paraventricular nucleus. Additionally, the intestinal sequestration of water and electrolytes triggers the release of angiotensin II in cases of paralytic ileus, which frequently occurs alongside acute episodes. Angiotensin II, via its receptors on the third ventricle floor, directly stimulates ADH release into the ventricle via baroreception [21] or hypothalamic supraoptic nucleus damage [22]. The elevation of plasma ALA and PBG levels is definitively the trigger for acute attacks in hepatic porphyrias; however, hyponatremia and the development of SIADH are critical factors influencing patient prognosis [21].

Atopic Dermatitis (AD)

Medical literature from Japan and other countries has reported case studies of newborns with severe AD who developed hyponatremia, often associated with hyperkalemia, despite elevated aldosterone levels [23,24]. The precise etiology of hyponatremia in severe infantile AD is not fully understood; however, increased sodium concentrations in skin exudates suggest that sodium depletion from compromised skin may be a contributing factor. The pathogenesis of this condition is similar to a rare form of pseudohypoaldosteronism (PHA) caused by mutations in the CA12 gene, which encodes carbonic anhydrase 12. The variant PHA causes sodium loss in the sweat glands. In contrast, classic PHA causes distal nephron aldosterone unresponsiveness due to mutations in the aldosterone receptor gene (NR3C2) or epithelial sodium channel genes (ENaC-a, -b, and -c) [25].

HIV/AIDS-Related Hyponatremia

**Figure 1:**
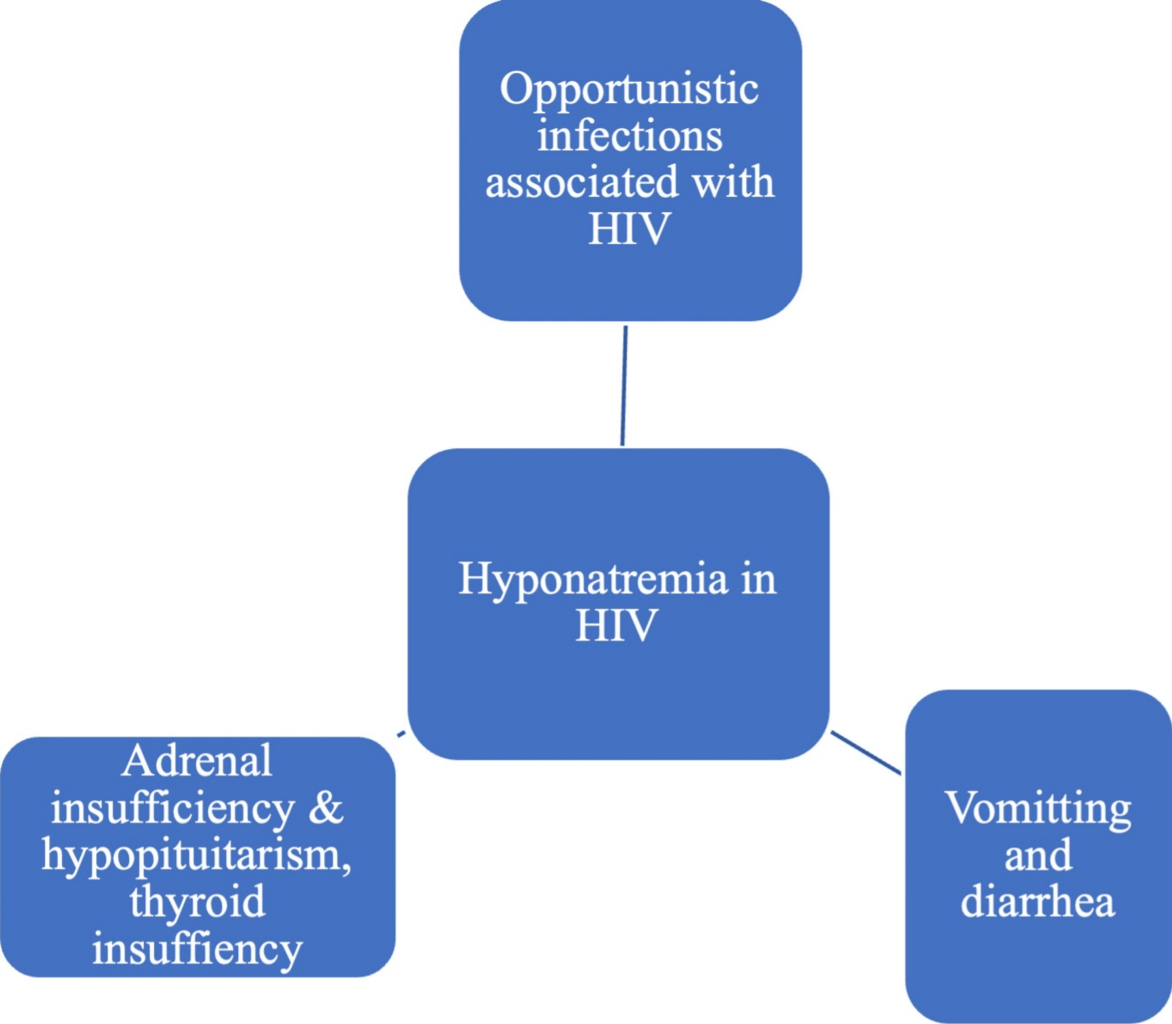
Flowchart showing the causes of hyponatremia in HIV. Reference: [26]. HIV, human immunodeficiency virus

Factors causing hyponatremia in HIV/AIDS are illustrated in Figure 1.

Abscesses, tuberculous meningitis, and encephalitis can cause increased ADH release. Various infectious agents, notably cytomegalovirus (CMV) in the necrotic foci, may account for the inflammation observed in the adrenal glands. HIV can induce inflammation, specifically of the medulla, due to the virus's affinity for the adrenal medulla, which originates from neural tissue and the neural crista. Deficiencies in aldosterone, cortisol, and adrenal androgens represent notable alterations. Hyponatremia results from hypovolemia and salt wasting due to deficits in both cortisol and aldosterone. The inadequate production of cortisol, which inhibits vasopressin release, is a notable factor contributing to hyponatremia. Numerous opportunistic infections in HIV, including Coccidioides, Pneumocystis jirovecii, Tuberculosis, and Cryptococcus, can affect systemic and thyroid function. A condition known as overt hypothyroidism occurs when the thyroid cannot produce and release sufficient T4 into the bloodstream despite stimulation by the thyroid-stimulating hormone (TSH). Hypothyroidism commonly presents with symptoms such as dry skin, hyponatremia, cold insensitivity, fatigue, altered voice, and constipation. Recurrent diarrhea and vomiting from HIV/AIDS-related opportunistic infections can cause hypovolemic hyponatremia due to extra-renal salt losses [26].

*Infections* 

Hyponatremia-related infections include murine typhus (Rickettsial), human herpesvirus 6 (HHV-6), Dengue, Hanta, Ebola (viral), and Cryptococcosis (fungal) [27,28].

Systemic Lupus Erythematosus (SLE)

Hyponatremia is infrequently observed in patients with lupus. In the clinical context of central nervous system (CNS)-related lupus activity, an increase in IL-6 production has been linked to dysregulated cerebral ADH secretion. This dysregulation is influenced by various stimuli that non-osmotically stimulate vasopressin, including inflammation and pain. Hyponatremia in lupus requires comprehensive investigation concerning its prevalence, underlying mechanisms, and association with disease activity. Limited cohorts in the literature indicate that hyponatremia may serve as a reflection of disease activity and could also correlate with markers of acute inflammation. A study by Yamany et al. indicates that hyponatremia may act as a marker for the activity of SLE. Additionally, it may function as a valuable prognostic marker of inflammation that is readily identifiable [29].

Miliaria Rubra

PHA type 1 (PHA1) is an uncommon autosomal recessive disorder defined by salt-wasting, hyperkalemia, metabolic acidosis, and aldosterone nonresponsiveness. This condition results in an abnormal and excessive loss of sodium chloride via urine, perspiration, and saliva, among other secretions. The skin lesions resulting from elevated salt levels in perspiration are similar to those observed in miliaria rubra. Few reports exist on PHA1 patients presenting with similar cutaneous eruptions [30,31].

Dermatological Drugs Causing Hyponatremia

Drugs causing hyponatremia are narrated in Table 2.

**Table 2:** Dermatological drugs causing hyponatremia. References: [32-43]. ENaC, epithelial sodium channels; Na⁺, sodium channel; K⁺, potassium channel; ICF, intracellular fluid; ECF, extracellular fluid; MAOIs, monoamine oxidase inhibitors; SSRIs, selective serotonin reuptake inhibitors; SNRIs, serotonin-norepinephrine reuptake inhibitors; TCAs, tricyclic antidepressants; SIADH, syndrome of inappropriate antidiuretic hormone; HDAC, histone deacetylase; MAPK, mitogen-activated protein kinase

Class	Drugs causing hyponatremia	Cause
Antibiotics	Trimethoprim-sulfamethoxazole (TMP-SMX)	TMP and amiloride are structurally similar and act on the same distal nephron epithelial ENaC, causing natriuresis and hyponatremia [32].
Diuretics	Spironolactone	Spironolactone reduces the aldosterone-induced formation of epithelial Na+ channels, improving Na+-K+ exchange and natriuresis [33].
Intravenous immunoglobulin (IVIG)		IVIG-induced pseudo hyponatremia is triggered by high plasma protein or fat levels, although plasma water sodium remains normal [34,35]. A case report describes a patient with impaired urinary free water excretion who developed true hyponatremia due to a significant infusion of diluted fluids and a sucrose-induced shift of water from ICF to ECF compartment [36].
Colchicine overdose		Colchicine intoxication damages proximal tubules, leading to renal failure and resulting in hyponatremia, hypomagnesemia, hypophosphatemia, and hypocalcemia [37].
Anticonvulsants	Carbamazepine and oxcarbazepine	SIADH is the principal cause of renal water retention, leading to hyponatremia [32].
Antidepressants	MAOIs, SSRIs, SNRIs, TCAs, mirtazapine, sertraline, and fluvoxamine	SIADH is the principal cause of renal water retention, leading to hyponatremia [38].
Chemotherapeutic agents	Vincritine, vinblastine, cisplatin, carboplatin, cyclophosphamide, and ifosfamide	SIADH and renal salt-wasting syndrome [39].
Antimicrobial	Topical silver nitrate	Silver nitrate induces cation leaching, leading to hyponatremia [40].
Others	Vismodegib [41], Pabinostat (HDFAC inhibitor) [42], Cobimetinib (MAPK inhibitor) [43].	

Hypernatremia

Hypernatremia is defined as a serum sodium content of more than 145 mmol/L, which is indicative of serum hyperosmolality, an electrolyte imbalance rare in dermatology. Hypernatremia can result from excessive salt consumption, substantial water deprivation, or heightened loss of free water [44]. Hypernatremia can lead to a spectrum of symptoms, including weakness, thirst, hyperreflexia, neuromuscular excitability, lethargy, coma, and seizures [45]. Dermatological conditions associated with hypernatremia are enumerated next.

Icthyotic Group of Disorders (Netherton Syndrome, Lamellar Syndrome, Collodion Baby, Superficial Epidermolytic Ichthyosis, and Others)

Ichthyoses represent a group of genetic disorders characterized by atypical epidermal cornification. These conditions are marked by a disruption of the skin’s protective barrier and a notable increase in sensible water loss, up to six times greater than the insensible water loss observed in normal skin [46]. This results in hypernatremic dehydration, which is associated with severe implications. Extremely preterm newborns exhibiting elevated transepidermal free water loss demonstrate increased susceptibility to hypernatremia. Clinically, decreased weight, tachycardia, and reduced output are observed [47]. Elevated serum sodium levels may lead to convulsions. Serum sodium levels exceeding 160 mmol/L can result in mortality. In such instances, an appropriate adjustment of free water is administered. Humidified incubators decrease water loss in these patients. Patients exhibiting hypernatremia with normal or elevated total body weight may require an alternative treatment approach for hypovolemic hypernatremia. Treating ichthyosis with increased fluid and normal sodium levels may be more beneficial than addressing hypernatremia with substantial quantities of both fluid and sodium. Furthermore, skin care with humidification and emollients lowers sensible water loss in patients, reducing hypernatremia and dehydration [47].

Erythroderma

Erythroderma, regardless of its etiology, impacts the skin of extensive areas, leading to significant transdermal fluid loss [48]. A study by Namdar et al. suggests an increased mortality risk in TEN patients who encounter in-hospital acquired hypernatremia. Therefore, immediate action is necessary [49]. However, Bastuji-Garin et al. conducted a multivariate analysis that did not establish a hypernatremic state as an independent risk factor for mortality. Therefore, serum sodium imbalance was excluded from the SCORTEN (Severity-of-Illness Score for Toxic Epidermal Necrolysis) calculation [50].

Psoriasis

Various exogenic factors that incite psoriasis and exhibit prooxidative effects have been identified, including smoking, alcohol consumption, narcotics use, infections, psychological stress, and physical injuries. Keratinocytes have Na+ (Sodium ion) selective channels, including the epithelial sodium channel and the Na+ permeable nonselective transient receptor potential, along with other cation channels that regulate their differentiation and inflammatory responses. Extracellular sodium concentration influences neuronal excitability and signal amplitude associated with psoriatic inflammation [51]. Psoriasis is marked by elevated reactive oxygen species (ROS) production and an impaired redox equilibrium. Maifeld et al. demonstrated in an observational study that skin Na+ levels outweigh serum levels in psoriasis patients with a psoriasis area and severity index (PASI) greater than 5. A high-salt environment can enhance the proliferation of pathogenic T-helper 17 cells in psoriasis. The data of this study were collected using a noninvasive method, specifically, sodium (23Na) magnetic resonance imaging. Subsequently, validation was conducted through 23Na spectroscopy and atomic absorption spectrometry in ashed-skin biopsies from patients and rodent models of psoriasis [52].

Autoimmune Disorders

Evidence indicates that elevated dietary salt intake leads to immune system dysregulation by facilitating the polarization of naive CD4+ T cells into IL-17-secreting T helper (Th) cells (Th17 cells). This process involves serum glucocorticoid kinase-1 (SGK1), which enhances the expression of the IL-23 receptor (IL-23R). IL-23/IL-23R plays a critical role in the differentiation of pathogenic inflammatory Th17 cells, which contributes to the initiation and maintenance of inflammation in autoimmune diseases such as SLE and psoriasis [53]. Hernandez et al. presented new evidence indicating that excessive salt markedly diminishes the functional activity of thymus-derived regulatory T (tTreg) cells. However, it did not alter Foxp3 (Forkhead box protein 3) expression [54]. The findings suggest a novel pathogenic mechanism in the development of psoriasis and identify a potential target for treatment. The exact role of dietary salt intake in the causation of immune-mediated diseases in the human population remains ambiguous [55]. It necessitates additional research, particularly clinical trials examining nutritional interventions that compare low- and high-salt diets. The interaction of salt with various environmental factors and its genetic origins presents a significant avenue for future research to enhance the assessment of the specific role of sodium chloride in the pathogenesis of immune-mediated diseases.

Miliaria Crystallina

There are a few documented cases of miliaria crystallina caused by hypernatremia in the literature. A case report by Chao reported an afebrile hypernatremic adult patient presenting with miliaria crystallina, suggesting that this condition may have resulted from the direct drying of corneocytes due to elevated salt levels [56]. Engür et al. [57] and Aydin et al. [58] documented cases of diffuse miliaria crystalline in neonates admitted to the NICU for acute hypernatremic dehydration who subsequently developed widespread and extensive miliaria crystallina following treatment. It was proposed that a potential mechanism may involve the degradation of sweat ducts, leading to the excretion of sweat with elevated salt concentrations.

Burns

Namdar et al. proposed that an electrolyte shift characterized by hypernatremia may signify hypovolemia, potentially leading to reduced tissue perfusion and subsequently impacting the healing process of burn wounds. The findings indicate that additional research is required to validate that hypernatremia serves as an indicator of dehydration and affects skin graft viability [59]. Stewart et al. [60] reported that burn sizes were significantly larger in individuals exhibiting severe hypernatremia (sodium > 150 mmol/L). This requires the establishment of protocols to enhance fluid volume following burn shock treatment [60]. A study by Sedghiani et al. indicates that patients with burns in a hypernatremic state experience higher mortality rates and prolonged hospital stays [61].

Salting of Skin

Limited reports of hypernatremia following skin salting have been reported in the literature [62,63]. The Turkish cultural practice of *salting*, involving the application of salt to the skin, is thought to improve the health of a newborn’s skin and reduce the odor of sweat [62]. Prolonged exposure to salt can lead to epidermolysis, a condition characterized by the burning of the outer layers of the skin, resulting in the absorption of salt through the epidermis and an increase in serum sodium levels [63].

Potassium

Potassium is the most prevalent cation in intracellular fluid. Potassium cations are abundant in ICF, mostly in muscles. However, only around 2% of the potassium in the body is found in the ECF. Plasma potassium concentrations typically vary between 3.5 and 5.0 mEq/L. Several factors, including potassium intake, renal excretion, loss through the alimentary canal, and hormones, including insulin, adrenaline, aldosterone, and glucocorticoids, affect potassium homeostasis. An electrical gradient produced by the sodium and potassium pump (Na+-K+-ATPase) is essential for both muscle contraction and nervous system function [64].

Hypokalemia 

Hypokalemia, a blood potassium level below 3.5 mEq/L, is a prevalent electrolyte disorder in clinical settings. Insufficient daily potassium consumption, severe potassium depletion, transcellular shifts in the abrupt movement of potassium from ECF to intracellular fluid, and specific medications can induce hypokalemia. Hyperaldosteronism can lead to significant hypokalemia (serum potassium < 3.0 mEq/L) and metabolic alkalosis. A correlation exists between hypokalemia and Cushing’s syndrome [65]. The most common symptoms upon presentation are fatigue and muscular weakness. Although rare, flaccid paralysis can occur due to severe hypokalemia-related muscle weakness. Muscle spasms are noted in certain patients. Rhabdomyolysis may occur due to significant hypokalemia. Constipation and ileus may arise from the involvement of the gastrointestinal musculature. Hypokalemia can also manifest clinically as palpitations, as well as arrhythmias and cardiac failure. Flat T waves, ST segment depression, and prominent U waves on the ECG are the defining characteristics of hypokalemia [66]. Dermatological diseases and medications associated with hypokalemia are discussed next.

Psoriasis

Research indicates that the vegetarian diet may alleviate the symptoms of psoriasis. The improved eicosanoid profile of a vegetarian diet significantly suppresses the inflammatory processes involved in psoriasis. However, the relationship between potassium deficiency and psoriasis requires further investigation [67]. A study by Lewinn et al. [68] employed a combination of extract of the adrenal cortex, improved vitamin C intake, and a diet with low potassium to reduce the progression of psoriasis. However, this study was unsuccessful as it failed to detect any impairment in adrenal cortical function. The *Cortisol-Potassium* theory was proposed to explain the advantageous effects of vegetarian diets, as evidenced by a clinical trial conducted by Rastmanesh [69]. This theory suggests that the improvement in psoriasis associated with vegetarian diets may be partially linked to increased dietary potassium intake, which, in turn, enhances cortisol biosynthesis and secretion.

Ectopic Cushing’s Disease as a Paraneoplastic Syndrome

Elevated cortisol levels in Cushing’s disease exceed the oxidative capacity of 11 beta-hydroxysteroid dehydrogenase, leading to the inactivation of cortisol to cortisone, which subsequently results in hypokalemia, a prevalent characteristic of Cushing’s syndrome. Hypercortisolism activates mineralocorticoid receptors in renal tubules, resulting in heightened mineralocorticoid activity, which subsequently leads to hypernatremia, hypokalemia, and metabolic alkalosis [70]. Metastatic MCC [71] and malignant melanoma [72] have been reported to manifest with hypokalemia, hypernatremia, hypertension, and hyperglycemia as a result of ectopic Cushing's syndrome.

HIV

Hypokalemia is observed in approximately 19% of patients with AIDS. The primary cause of hypokalemia is gastrointestinal potassium loss, often resulting from diarrhea associated with intestinal infections, tumors, or enteropathy related to AIDS. Vomiting also induces hypovolemia, bicarbonaturia, and secondary hyperaldosteronism, which increases the quantity of potassium excreted in the urine, in addition to causing direct potassium loss (emesis). Furthermore, urinary potassium depletion may coexist with tubule injury that is the direct result of the toxic effects of nephrotoxic medications (such as aminoglycosides and amphotericin B), specific antibiotics (such as cephalosporins and sulfonamides), or non-steroidal anti-inflammatory drugs (NSAIDs) used in HIV [73].

Drug-Induced Hypokalemia

Drugs causing hypokalemia are listed in Table 3.

**Table 3:** Drugs causing hypokalemia. References: [74-88] Na-K-ATPase, sodium-potassium pump; RTA, renal tubular acidosis; mTOR, mechanistic (or mammalian) target of rapamycin; HDACi, histone deacetylase inhibitor; PD-1, programmed death-1; EGFR-1, epidermal growth factor receptor-1; TRPM6/7, transient receptor potential cation channel subfamily M member 6/7

Class	Drugs causing hypokalemia	Mechanism
Immunosuppressives	Systemic corticosteroids	Corticosteroid-induced insulin resistance may result in hyperglycemia and hyperinsulinemia. Insulin promotes the intracellular shift of serum potassium by enhancing the quantity and activity of Na-K-ATPases [74].
Antibiotics	Penicillin	Penicillin, a non-absorbable anion, causes hypokalemia by inducing the distal renal tubule to absorb more sodium than potassium. A lumen-negative gradient is maintained by volume depletion, which increases aldosterone and decreases distal chloride transport [75].
	Aminoglycosides	Aminoglycosides may lead to magnesium deficiency, subsequently resulting in renal potassium wasting. The mechanism by which aminoglycosides induce magnesuria and kaliuria remains incompletely understood. This may result from tubular toxicity and hyperaldosteronism caused by aminoglycosides [75].
	Other antibiotics (Azithromycin, Ciprofloxacin, Piperacillin-tazobactam, Colistin, Vancomycin, meropenem and rifampicin) [76]	
Antifungals	Amphotericin B [75]	Amphotericin B induces nephrotoxicity, damaging the renal tubules and subsequent renal potassium wasting. Moreover, magnesium depletion results in the inhibition of hydrogen ion secretion by secretory duct cells. The resultant hypokalemia is dose-dependent and often precedes renal failure.
	Itraconazole [77], Flucanozole [78]	These medications induce the urinary excretion of potassium, leading to pronounced hypokalemia.
	Posaconazole [79]	Posaconazole inhibits the enzyme 11β-hydroxylase, resulting in elevated levels of the mineralocorticoid receptor activator deoxycorticosterone.
Antivirals	Acyclovir [80]	Acyclovir can cause hypokalemia through crystal-induced damage to the distal tubules and diminished renal plasma flow.
	Foscarnet [81]	
	Tenofovir [82]	Tenofovir-induced type 2 RTA increases renal bicarbonate excretion. The distal nephron exhibits elevated intraluminal negativity due to enhanced bicarbonate delivery, leading to potassium secretion and subsequent wasting.
Tyrosinase inhibitors	Imatinib [83]	Multiple studies have shown that after severe kidney damage, the drug imatinib inhibits renal tubulogenesis, also known as tubular regeneration, by lowering the number of active tubular cells. This, consequently, prevents potassium from being absorbable from the glomerular filtrate. Potassium that is not reabsorbed will be excreted through the urine; consequently, blood potassium levels may decrease. As long as imatinib inhibits renal tubule regeneration, reabsorption will be disrupted.
mTOR inhibitor	Sirolimus [84]	Sirolimus induces mild tubular dysfunction, increasing potassium excretion in the urine and subsequent hypokalemia.
HDACi	Panobinostat [85]	
PD-1 inhibitor	Pembrolizumab [86]	Pembrolizumab lowers the ability of the kidneys to retain potassium.
Monoclonal antibodies to EGFR-1 or HER-1	Cetuximab and Panitumumab [87]	EGFR prevents TRPM 6/7 ion channels from reaching the apical membrane of the distal tubule, regulating magnesium reabsorption. These medications inhibit apical TRPM ion channels through EGFR blockade, leading to hypomagnesemia and renal magnesium wasting, which may result in hypokalemia and cardiac arrhythmias.
Salicylate intoxication [88]		Salicylates initially modify the acid-base equilibrium by inducing respiratory alkalosis. The anterior hypothalamus is crucial in mediating respiratory alkalosis through direct central influence. Hypokalemia is a notable consequence of respiratory alkalosis.
Colchicine overdose [37]		

Hyperkalemia

Hyperkalemia, characterized by serum potassium levels exceeding 5.5 mmol/L, is potentially fatal. High potassium consumption, decreased kidney excretion, or potassium leakage associated with disorders, including rhabdomyolysis, hypoaldosteronism, and acute and chronic renal failure, are some causative factors. Symptoms of hyperkalemia are infrequently observed, although individuals may encounter nausea, palpitations, myalgia, or arrhythmia. Nevertheless, moderate to severe hyperkalemia may lead to potentially lethal cardiac arrhythmias. ECG abnormalities include ST-segment depression, elevated T-waves, and QRS widening [89]. Diseases and medications used in dermatology associated with hyperkalemia are discussed next.

HIV

A few reports of hyperkalemia in HIV-positive individuals have been documented in the literature. Adrenal insufficiency [90], hyporeninemic hypoaldosteronism [91], medications such as trimethoprim [92] or pentamidine [93], and impaired kidney function due to HIV-induced kidney disease are mechanisms contributing to the development of hyperkalemia. The mechanism responsible for the diminished release of renin in HIV-positive patients is not yet understood, nor is the impact of direct adrenal damage on the decreased release of aldosterone clarified. Mineralocorticoid replacement therapy with fludrocortisone (0.1-2 mg daily) effectively addresses hyperkalemia resulting from hypoaldosteronism [94].

SLE

Brentjens et al. postulated that renal tubular resistance to mineralocorticoids exists in patients with SLE, influencing potassium secretion. Deposits of antigen-antibody complexes in the basement membrane of tubules and interstitial tissue interfere with distal tubular function, resulting in decreased potassium secretion and subsequent hypokalemia [95]. Currently, hyporeninemic hypoaldosteronism is regarded as the primary pathomechanism underlying hyperkalemia in SLE [96]. Furthermore, the overuse of nonsteroidal anti-inflammatory drugs (NSAIDs) may worsen potassium handling impairment in patients with SLE. These factors are associated with the induction of hyporeninemic hypoaldosteronism due to their role in decreasing prostaglandin synthesis, subsequently impacting renin synthesis [97].

Drug-Induced Hyperkalemia 

Drugs causing hyperkalemia are narrated in Table 4.

**Table 4:** Drugs causing hyperkalemia. References: [32,33,89,98-108] Na⁺, sodium; K⁺, potassium; CCBs, calcium channel blockers; KATP, ATP-sensitive potassium channel; COX-2, cyclooxygenase-2; TMP-SMX, trimethoprim-sulfamethoxazole; ENaC, epithelial sodium channel; KI, potassium iodide; KOR, kappa opioid receptor; TLS, tumor lysis syndrome

Class	Drugs causing hyperkalemia	Cause
Diuretics	Spironolactone	Spironolactone and its active metabolites act as aldosterone antagonists by competitively binding to receptors at the sodium-potassium exchange sites in the distal convoluted tubule of the renal system [33].
Beta-blockers	Oral Propronolol [98,99] and topical Tomolol [100,101]	The postulated theories of β-blocker-induced hyperkalemia include inhibition of renin production, disruption of intracellular potassium uptake, and promotion of endothelial cell apoptosis, which releases intracellular potassium [99].
ACE inhibitors, Angiotensin-II receptor antagonists		Reduced adrenal aldosterone production due to a blockage of the renin-aldosterone axis results in hyperkalemia [89].
Calcium channel blockers (CCBs)	Nifedipine, Amlodipine	CCBs limit the synthesis of adrenal aldosterone [89].
Immunosuppressants	Cyclosporin	Proposed mechanisms of hyperkalemia induced by Cyclosporine are hyporeninemic hypoaldosteronism, inhibition of apical secretory K+ channels in the distal collecting tubule resulting in decreased kaliuresis, and disruption of basolateral Na+/K+-ATPase activity. They partially inhibit COX-2 production in the macula densa. Distributive hyperkalemia represents a notable side effect of cyclosporin, particularly in conjunction with β-blockers. A recent report indicates that the ability of Cyclosporine to open KATP channels, which are widely distributed in vascular smooth muscle, may lead to hyperkalemia [89].
Antibiotics	Penicillin	When administered as a potassium salt, the potassium load in penicillin is probably the cause of hyperkalemia [102].
	Trimethoprim-sulfamethoxazole (TMP-SMX)	Trimethoprim (TMP) and amiloride exhibit structural similarities, and both act on the distal nephron epithelial sodium channels (eNAC), leading to natriuresis, hyponatremia, and reduced potassium secretion. resulting in hyperkalemia [32].
NSAIDs		NSAIDs restrict the synthesis of aldosterone induced by angiotensin II and reduce renin secretion, partially regulated by locally produced prostaglandins. The simultaneous decrease in aldosterone secretion will result in diminished potassium excretion in the urine, consequently causing hyperkalemia [103].
Anticoagulants	Heparin	Heparin inhibits the synthesis of adrenal aldosterone and diminishes the number and affinity of angiotensin-II receptors [89].
Potassium iodide (KI)		No case reports of hyperkalemia exist when used alone. However, KI should be avoided in cases such as chronic renal failure and in patients using potassium-sparing diuretics or angiotensin-converting enzyme inhibitors to prevent hyperkalemia [104].
Androgen receptor inhibitor	Clascoterone	Clascoterone and spironolactone share structural similarities, which accounts for hyperkalemia. The shift from normal to increased potassium levels was infrequently reported in both Phase I and II trials of clascoterone. However, the association between clascoterone and a significant risk of hyperkalemia remains unclear. Hyperkalemia was occasionally noted in both clascoterone- and vehicle-treated patient groups. However, an exposure-response analysis indicated no correlation between hyperkalemia and plasma levels of clascoterone or its metabolite, cortexolone. The incidence of elevated potassium levels was most significant among patients under twelve years of age receiving clascoterone treatment, in whom 1% of clascoterone lacks FDA approval [105].
kappa opioid receptor (KOR) agonist	Difelikefalin (first and only FDA-approved treatment for uremic pruritus) [106]	
Drug-induced tumor lysis syndrome (TLS)	Chemotherapeutic agents (cytarabine, cisplatin, etoposide, and paclitaxel) [107]	TLS represents a metabolic disturbance resulting from the apoptosis of neoplastic cells during cancer therapy, releasing intracellular constituents into circulation. Hyperkalemia often represents an early manifestation of TLS, as potassium may start to leave dying neoplastic cells before their lysis. Tumor lysis syndrome (TLS) is marked by the abrupt occurrence of hyperkalemia, hyperuricemia, hyperphosphatemia, and hypocalcemia, which can be life-threatening [107].
	Rituximab (when used in nondermatological conditions like malignancy) [108]	

Calcium

Calcium (Ca+2) is a divalent cation crucial for multiple physiological functions, such as blood coagulation, signal transduction, neural conduction, skeletal mineralization, and muscular contraction [109]. The usual range of serum Calcium is 8.8-10.4 mg/dL [110]. Phosphate and Magnesium (Mg+2) metabolism are interconnected with calcium metabolism. Reabsorption from the renal tract, intestinal tract, and bone turnover are essential for sustaining Ca+2 homeostasis. The hormones PTH, calcitriol, and serum ionized Ca+2 modulate calcium-mediated processes [109].

Hypocalcemia

Hypocalcemia can be attributed to several factors, including vitamin D deficiency, hypoparathyroidism, hyperphosphatemia, hypomagnesemia, and certain medications [111]. It may present as either acute or chronic. The severity of hypocalcemia influences its clinical presentation. Clinical presentation includes fatigue, muscular weakness, disorientation, depression, and amnesia [112]. Acute hypocalcemia may lead to significant symptoms, including tetany, anxiety, laryngospasm, paresthesias, seizures, and QT interval prolongation. Chvostek’s sign and Trousseau’s sign may be observed in patients with hypocalcemia, resulting from increased neuromuscular excitability. Trousseau’s sign is a carpopedal spasm resulting from forearm ischemia when the cuff is inflated above the systolic blood pressure for three minutes during blood pressure assessment. Chvostek’s sign elicits twitching in the facial muscles when the area along the jaw angle, approximately 2 cm anterior to the earlobe, is tapped [113]. Chronic hypocalcemia may manifest as ridging over nails, coarse and brittle hair, and dry, keratotic skin [114]. Dermatological conditions and drugs associated with hypocalcemia are discussed below.

Psoriasis

Patients diagnosed with Psoriasis vulgaris, Pustular psoriasis of Von Zumbusch, and Impetigo herpetiformis can experience hypocalcemia [115]. Hypocalcemia is associated with Impetigo herpetiformis and is considered a secondary metabolic condition. The extensive cutaneous inflammation leads to the extravasation of albumin and albumin-bound calcium into the interstitial space, resulting in hypoalbuminemia and subsequent hypocalcemia [116]. Wolf et al. [117] also reported a case of compensating hyperparathyroidism in a patient with severe impetigo herpetiformis, indicating that exudative hypoproteinemia, which was also present in the patient, causes loss of free and protein-bound serum calcium. Low-normal calcium levels are due to secondary hyperparathyroidism, compensating for calcium loss from the cutaneous exudation. The parathyroid hormone (PTH)-induced conversion of 25-OH (hydroxy) vitamin D3 to 1,25-OH vitamin D3 may also explain the increased uptake of 25-OH vitamin D3 [117]. Calcium homeostasis may play a role in the initiation or worsening of psoriasis, as hypocalcemia may affect cell adhesion molecules [118]. Cadherins are calcium-dependent protein molecules essential for cell adhesion. Calcium plays a significant role in the proliferation and differentiation of keratinocytes [119]. The exact mechanism underlying the relationship between calcium and keratinocyte differentiation remains unclear; however, these pathophysiological factors may elucidate the potential link between hypocalcemia and psoriasis flare-ups.

Acute generalized pustular psoriasis (GPP) accompanied by hypocalcemia has been reported in case studies where hypoparathyroidism was identified as the underlying cause [115,120]. Impetigo herpetiformis has also been associated with hypocalcemia of congenital rickets [121]. The diminished intestinal absorption of vitamin D has been etiologically linked to impetigo herpetiformis [122]. A study by Maheswari and Dutta noted a significant association between hypocalcemia and pustular psoriasis, stressing the importance of maintaining calcium levels to prevent the progression of psoriasis. Additionally, a moderately strong negative correlation was noted between serum calcium levels and the severity of psoriasis [123]. The restoration of calcium in relation to vitamin D is an essential part of the acute GPP treatment if hypocalcemia is identified [124,125]. Thus, dietary supplementation may prevent the disease’s progression to more advanced stages and enhance the prognosis. This conclusion aligns with the study by Ilyas et al. [126], which found that a significant proportion of patients with chronic plaque psoriasis exhibited low serum calcium levels. Hypocalcemia may indicate the severity of psoriasis [126]. Hence, routine screening of serum calcium levels in psoriasis patients should be employed, as it facilitates quicker diagnosis and the early identification and treatment of associated complications. 

Fahr’s syndrome, or bilateral striopallidodendate calcinosis, is a rare neurological disorder often presenting asymptomatically with bilateral cerebral calcifications, which may be associated with hypocalcemia [127]. There are reports of severe hypocalcemia associated with GPP and Fahr’s syndrome [128,129]. A study by Zhai et al. indicated that elevated tCa (total calcium) levels correlated with enhanced MTX (methotrexate) efficacy. MTX inhibits keratinocyte growth, with calcium enhancing this effect synergistically [130].

Ichtyotic Group of Disorders

The following factors are primarily responsible for the association between ichthyosis and rickets:

(1) Increased keratinocyte proliferation leads to increased epidermal thickness, thereby diminishing sunlight penetration.

(2) Vitamin D-dependent rickets results from insufficient vitamin D synthesis in the compromised epidermis.

(3) Elevated calcium loss occurs via epidermal desquamation with limited sun exposure due to associated intolerance to heat.

(4) The use of systemic retinoids inhibits calcium absorption from the gastrointestinal tract.

The factors discussed above enhance PTH secretion, elevating the risk of rickets in children with ichthyosis [131,132]. Literature reports indicate an association between rickets and conditions such as ichthyosis vulgaris, lamellar ichthyosis, epidermolytic hyperkeratosis, X-linked ichthyosis, and non-bullous ichthyosiform erythroderma [133,134,135].

Hypocalcemic vitamin D-resistant rickets (HVDRR) is caused by a defect in the vitamin D receptor rather than by a deficiency of vitamin D. The case report by el-Khateeb presents a rare association between bullous congenital ichthyosiform erythroderma and HVDRRs. Bullous congenital ichthyosiform erythroderma is a rare autosomal dominant condition characteristic caused by spontaneous mutations in the keratin 1 (KRT1) gene on chromosome 12 [12q13] and/or 17 [17q21-22]. Mutations in the VDR gene on chromosome 12 [12q13-14] cause autosomal recessive HVDRR. The close genetic proximity of these disorders on the long arm of chromosome 12 may indicate a common etiology, although coincidence remains a possible explanation for this association [136].

Conradi-Hünermann-Happle (CHH) syndrome is a rare X-linked dominant disorder characterized by blaschkoid ichthyosiform scaling, follicular atrophoderma, congenital cataracts, and stippled epiphyseal calcifications. The association between CHH syndrome and severe hypocalcemia in newborns has been documented in several case reports in the literature [137,138,139]. It was postulated that transitory tissue deposition caused hypocalcemia and hypoparathyroidism, characterized by elevated serum phosphorus and abnormal PTH levels [139].

A case study details a child with congenital ichthyosis presenting with hypoparathyroidism and hypocalcemia at six weeks of age, alongside sensorineural deafness identified at four years of age. Initial clinical testing was unable to ascertain the cause. Subsequently, novel mutations in GATA binding protein 3 (GATA3) and steroid sulfatase (STS) - responsible for hypoparathyroidism-sensorineural deafness-renal disease syndrome (HDR syndrome) and X-linked congenital ichthyosis, respectively - were identified through whole-genome sequencing (WGS) [140].

Pseudohypoparathyroidism (PHP) and pseudopseudohypoparathyroidism (PPHP) are both classified under Albright’s hereditary osteodystrophy (AHO). PHP and PPHP have identical characteristics, but patients with PHP do not respond to PTH and have hypocalcemia, while patients with PPHP have normal serum calcium levels. The condition is a clinical combination of physical characteristics, including short stature, rounded facial features, central obesity, brachydactyly, and impaired and delayed primary and secondary dentition, accompanied by varying degrees of intellectual disability and seizures. Existing literature has case reports of hypocalcemia linked to AHO with cutaneous bone Formation (osteoma cutis) [141,142,143].

Sturge-Weber Syndrome and Phakomatosis Pigmentovascularis With Dermal Melanocytosis

Neurovascular calcification, characterized by localized *tramtracking* on plain skull radiography and postnatal neurological deterioration, is a crucial indicator of GNAQ (G protein guanine nucleotide binding protein alpha subunit q)/GNA11 mosaicism. This finding is associated with conditions like Sturge-Weber syndrome and phakomatosis pigmentovascularis with dermal melanocytosis. A study by Knöpfel et al. [144]. demonstrated evident, though moderate, abnormalities in the calcium metabolic profile of the overall cohort, indicating a tendency towards ionized hypocalcemia. Normal parathyroid and renal function in the study suggested that antiseizure medicines, which produce 25-hydroxy-vitamin D insufficiency and lower calcium levels, could be the contributing factor. Levetiracetam and oxcarbazepine, two prominent seizure medicines, were associated with reduced calcium levels (although not necessarily abnormal). This is consistent with the findings of a recent study by Aksoy et al. [145] on (non-GNAQ/GNA11) patients with seizures. They further hypothesized that abnormal calcium fluxes in and around damaged blood artery foci might be linked to neurovascular calcification and affect serum calcium levels. Microvascular calcification may represent an overlooked component of the disease that can result in brain tissue hypoxia unrelated to leptomeningeal involvement. Also, an alternative rationale was suggested due to the limited number of patients exhibiting normal vitamin D levels. Calcium is recognized for its role in stabilizing excitable membranes; thus, reduced serum calcium levels may not directly induce seizures in healthy individuals, but they could serve as a contributing factor in patients with seizure disorders. Furthermore, local extracellular perivascular calcium levels may be markedly diminished due to the cellular abnormalities identified in the study by Zecchin et al. [146], potentially exerting direct local effects on adjacent cells near vascular malformations.

Necrotizing Fascitis

Hypocalcemia is commonly observed in critically ill patients and is, therefore, more recognized among critical care professionals. A case study by Nakamura et al. [147] demonstrated that the clinical worsening of necrotizing fasciitis was preceded by significantly lower serum calcium levels, suggesting that hypocalcemia indicates the severity of necrotizing fasciitis. Kranz et al. [148] stated that bacterial lipase leads to the degradation of subcutaneous fatty tissue in necrotizing fasciitis, producing free fatty acids that saponify ionized calcium. Similar findings were observed in the study by Guzmán-Aguilar [149], where hypocalcemia, along with other markers such as hypoprothrombinemia, anemia, and elevated creatine phosphokinase, demonstrated a statistically significant relationship with mortality in patients with necrotizing fasciitis. 

Immunodeficiency Syndrome

DiGeorge syndrome (DGS), also called 22q11.2 deletion syndrome (22q11.2DS), is a hereditary condition characterized by a hypoplastic thymus among its various components. Complete athymia, classified as a complete DiGeorge abnormality, occurs in a limited number of patients with DGS. One-third of these patients exhibit eczematous dermatitis, lymphadenopathy, and oligoclonal T-cells [150]. DiGeorge indicated that hypocalcemia results from either aplasia or hypoplasia of the parathyroid gland due to a developmental defect [151]. A study by Cheung et al. indicated that the higher incidence of hypocalcemia in 22q11·2DS is linked to primary hypoparathyroidism, reduced PTH reserve, hypothyroidism, and hypomagnesemia [152].

Transverse Leukonychia

Transverse leukonychia in all nails is associated with severe hypocalcemia, and calcium supplementation has demonstrated a positive effect. The proposed theories for the onset of leuconychia in hypocalcemic patients comprise the induction of digital arteriolar spasm and the disorganization of hard keratin in the nail [153]. Additional nail changes documented in hypocalcemia include longitudinal ridging of nails, brittle nails with onychorrhexis, hapalonychia, or softened nails [154,155]. 

HIV

Hypocalcemia in HIV infection is an uncommon phenomenon primarily associated with vitamin D insufficiency, hypoalbuminemia [156], the use of medications such as tenofovir [157] and foscarnet [158], and opportunistic infections. Furthermore, a case report of primary hypoparathyroidism exhibiting severe hypocalcemia symptoms has been associated with HIV infection [159]. Parathyroid cells may serve as a target for HIV because they express a CD4-like molecule, potentially leading to a reduction in PTH secretion [160].

Subcutaneous Fat Necrosis of the Newborn (SCFNN)

SCFNN is a rare, benign inflammatory condition affecting adipose tissue. The majority of cases of SCFNN were associated with hypercalcemia. Perinatal hypoxia, recognized as a risk factor for SCFNN, is believed to be causally linked to hypocalcemia via transitory functional hypoparathyroidism [161,162]. Minagawa et al. suggest that fetal hypoxia may have contributed to the onset of temporary pseudohypoparathyroidism and subsequent hypocalcemia by worsening the functional immaturity of the PTH receptor and the intracellular signal transduction pathway [163].

Chemical Injury

Hydrofluoric acid is a highly caustic substance employed for the manufacturing of graphite and ceramics, as well as in electropolishing, metal picking, cleaning solutions, insecticides, and laundry powder. Case reports have documented hypocalcemia and hypomagnesemia following hydrofluoric acid chemical injury. Hydrofluoric acid, upon contact with skin, can bind with fluoride anions, potentially leading to a considerable depletion of serum calcium and Mg [164,165,166].

Drug-Induced Hypocalcemia

Drugs resulting in hypocalcemia are detailed in Table 5.

**Table 5:** Drugs causing hypocalcemia. References: [167-176] PTH, parathyroid hormone; CYP450, cytochrome P450; PPIs, proton pump inhibitors; RANK, receptor activator of nuclear factor kappa B ligand; EGFR, epidermal growth factor receptor

Class	Drugs causing hypocalcemia	Cause
Immunosuppressives	Glucocorticoids	Glucocorticoids can disrupt calcium homeostasis but rarely lead to clinically significant hypocalcemia. Corticosteroids cause hypocalcemia by decreasing intestinal calcium absorption, inhibiting the conversion of cholecalciferol to 25-hydroxycholecalciferol, and increasing calcium protein binding due to steroid-induced alkalosis [167].
Antifungals	Amphotericin-B	Possible reasons are: (1) Hypomagnesemia due to amphotericin B results in reduced parathyroid hormone (PTH) secretion and increased resistance to PTH in bone tissue. (2) While the etiology remains unclear, low vitamin D levels (1,25-dihydroxy vitamin D) are commonly observed in individuals with hypomagnesemia, potentially resulting in hypocalcemia [168].
Antivirals	Foscarnet	Foscarnet-induced ionized hypomagnesemia may hinder preformed parathyroid hormone (PTH) excretion or cause target organ resistance, potentially resulting in ionized hypocalcemia [169].
Anticonvulsants	Phenytoin	Phenytoin, being a CYP450 inducer, accelerates the catabolic rate of 25-hydroxyvitamin D3 and 1α, 25-hydroxyvitamin D3 into inactive metabolites, leading to hypocalcemia [170].
Proton pump inhibitors (PPIs)		pH levels influence calcium absorption; thus, gastric achlorhydria caused by proton pump inhibitors (PPIs) reduces calcium bioavailability, leading to hypocalcemia [171].
Calcimimetic	Cinacalcet [172]	
RANK ligand inhibitor (inhibits the resorption of the bone by osteoclasts)	Denosumab [173]	
Bisphosphanates	Zoledronic acid [174]	
EGFR inhibitor	Cetuximab	PTH suppression due to severe hypomagnesaemia induced by cetuximab leads to hypocalcemia [175].
Rituximab (non-dermatological indication like tumor)		Hyperkalemia and hyperphosphatemia arise rapidly following tumor cell lysis, as potassium is the predominant cation and phosphate is the most abundant anion within cells. Elevated phosphorus levels in circulation are detrimental primarily due to their chelation of ionized calcium, leading to hypocalcemia, which can result in tetany, cramps, convulsions, arrhythmias, hypotension, and ultimately death [176].

Hypercalcemia

Hypercalcemia is characterized by calcium levels of more than 10.5 mg/dL. Hypercalcemia can be induced by the following conditions: hyperparathyroidism; malignancies that synthesize PTH-releasing peptide; ectopic production of PTH; extrarenal activation of vitamin D; medications such as thiazide diuretics, calcium, and lithium carbonate; vitamin A and D supplementation; and granulomatous diseases, including sarcoidosis and tuberculosis. The majority of patients with hypercalcemia are asymptomatic. A restricted number of patients exhibit the signs and symptoms enumerated next.

Muscular: Myalgia and Weakness 

Gastrointestinal: Nausea, emesis, and constipation

Cardiovascular: Bradycardia, tachycardia, atrioventricular block, prolonged PR interval, widened QRS complex, and shortened QTc interval

Renal: Dehydration, acute kidney injury, hyperuricemia, and nephrolithiasis

Neurological: Disorientation, lethargy, anxiety, cognitive impairment, behavioral changes, and coma [177]

Intravenous hydration, corticosteroids, agents that inhibit bone resorption, including denosumab and bisphosphonates, and, most necessarily, addressing the underlying cause have all been employed to manage hypercalcemia [178]. Dermatological conditions and medications linked with hypercalcemia are discussed next.

Subcutaneous Fat Necrosis (SCFN)

SCFN is a rare panniculitis that usually occurs in full-term babies following delivery trauma or hypoxia, aspiration of meconium, or therapeutic cooling [179]. Hypercalcemia is a potentially fatal adverse effect that impacts a small percentage of patients with SCFN. Recent statistics indicate that 45.6% of newborns with SCFN [180] exhibit hypercalcemia. SCFN-induced hypercalcemia may be asymptomatic or present with a wide array of symptoms. Therefore, the resulting hypercalcemia is likely to remain undiagnosed if SCFN is not clinically identified. Newborns may present with lethargy, irritability, poor feeding, failure to thrive, nausea, vomiting, and subsequent dehydration and hypotonia [181]. The pathophysiology of SCFN-related hypercalcemia remains incompletely understood; however, the following explanations have been proposed: 

(1) Increased expression of peripheral 1α-hydroxylase, along with a decrease in peripheral 24α-hydroxylase expression, leads to elevated blood levels of 1,25(OH)2 D3 (1,25-dihydroxyvitamin D3), subsequently resulting in higher serum calcium levels [181,182].

(2) Several studies indicate a direct release of calcium from necrotic skin patches in neonates with SCFN and associated hypercalcemia [183].

(3) Elevated levels of prostaglandin E2 in the urine in some patients with hypercalcemia indicate that increased calcium resorption from bone is a contributing factor to their hypercalcemia [184].

Calcinosis Cutis

Metastatic calcinosis cutis refers to the deposition of calcium due to hypercalcemia, which can occur in conditions such as parathyroid neoplasm, hypervitaminosis D, excessive milk and alkali intake, and significant bone destruction from osteomyelitis or metastatic carcinoma. It can also arise from hyperphosphatemia, commonly associated with chronic renal disease and secondary hyperparathyroidism [185].

Calciphylaxis is an uncommon and potentially fatal condition marked by medial calcification of small to medium-sized cutaneous arteries, clinically manifesting as severe and progressive cutaneous ulcerations. While documented exceptions exist, individuals undergoing dialysis or recovering from a renal transplant commonly suffer from calciphylaxis. Patients with HIV infection are at increased risk for renal failure, which may manifest as a severe complication [186]. The pathophysiology of calciphylaxis remains incompletely understood; however, factors such as elevated calcium-phosphate product, increased serum levels of PTH, activated vitamin D treatment, medications such as warfarin, iron therapy, and corticosteroids have been associated with it. The primary management involves surgical debridement of the wound, systemic antimicrobials, elimination of potential causes of ectopic calcification, and the use of chelating agents like sodium thiosulfate, cinacalcet, and hyperbaric oxygen therapy [187].

Granulomatous Disorders

Granulomatous diseases and dysregulated calcium homeostasis are recognized to be interconnected [188]. Around 10% of patients with sarcoidosis exhibit hypercalcemia. Hypercalcemia in sarcoidosis is attributed to macrophages producing 1,25(OH)2 D3 in an uncontrolled manner, leading to enhanced calcium absorption in the gastrointestinal tract and increased calcium resorption in the bone [189]. There are few reports concerning hypercalcemia attributed to PTH-related protein (PTHrP) in sarcoidosis and other granulomas [190,191,192]. Other granulomatous disorders, including tuberculosis [193], leprosy [194], disseminated candidiasis [195], disseminated histoplasmosis [196], and cat scratch disease [197], have also been associated with hypercalcemia. A case report detailing hypercalcemia in an immunocompetent patient with disseminated Mycobacterium marinum infection, with a rain barrel identified as the probable primary source, has also been reported [198].

Foreign Body-Induced Granulomas

The introduction of either organic or inorganic substances, such as silicone, can cause a foreign body reaction in the immune system, resulting in chronic granulomatous inflammation. As a result, silicone granulomas (also known as siliconomas) can cause hypercalcemia, a rare but potentially dangerous condition [199]. Case reports of hypercalcemia following silicone injections [200], silicone breast implants [201], and topical liquid silicone combined with transdermal ultrasonography for wrinkle reduction [202] are reported. Furthermore, there is an atypical case of hypercalcemia that may be associated with palisaded neutrophilic and granulomatous dermatitis as a novel etiology of hypercalcemia (PNGD). This hypercalcemia is hypothesized to result from elevated 1α-hydroxylase expression in the granuloma and epidermal cells in PNGD [203].

Polymethylmethacrylate (PMMA) is a commonly used injectable filler for cosmetic applications; however, its approval is limited to minimally invasive facial tissue procedures. PMMA injections are associated with chronic renal damage and hypercalcemia, which arise from a granulomatous foreign body reaction leading to extrarenal calcitriol synthesis [204,205].

Hypercalcemia of Malignancy

Patients with advanced-stage malignancies frequently exhibit hypercalcemia. Hypercalcemia of malignancy is characterized by elevated calcium levels, leading to significant symptoms. The postulated causes are mentioned as follows:

(1) PTHrp and PTH exhibit structural similarities. PTHrp interacts with the identical PTH bone receptors, stimulating osteoclast activity and facilitating the production of receptor activator of nuclear factor-kappa B ligand (RANKL), which subsequently causes resorption of the bone, releasing calcium into the bloodstream. PTHrp additionally facilitates calcium reabsorption through renal tubules. PTHrp does not enhance vitamin D hydroxylation in the kidneys or increase intestinal calcium absorption, unlike PTH [206].

(2) Approximately 20% of malignancy-related hypercalcemia is attributed to osteolytic metastases and the excessive calcium release from bone. Inflammatory cytokines such as interleukin (IL)-1, IL-3, IL-6, tumor necrosis factor-alpha (TNF-α), and transforming growth factor-beta (TGF-β) are produced by metastases. These cytokines stimulate osteoblasts to produce RANKL, which activates osteoclasts, leading to calcium resorption from bone and its release into the extracellular fluid [206].

(3) Ectopic hyperactivity of 1-alpha-hydroxylase leads to the formation of calcitriol (1,25-dihydroxycholecalciferol) [206].

(4) Ectopic production of PTH [206].

Dermatological malignancies presented with hypercalcemia are narrated in Table 6.

**Table 6:** Skin tumors and hypercalcemia. References: [207-223]. SCC, squamous cell carcinoma

Malignancy associated with hypercalcemia	Origin
Primary Cutaneous SCC [207]	Hidradenitis suppurativa [208,209] Sacral decubitus ulcer [210]
Genital SCC (Buschke-Löwenstein tumor) [211,212]	Giant condyloma acuminatum
Metastatic melanoma [213,214]	-
Melanoma	Congenital giant pigmented nevus [215]
Melanoma is associated with parathyroid adenoma and Primary hyperparathyroidism [216,217]	-
Cutaneous T-cell lymphoma [217], CD30-negative cutaneous large-cell lymphoma [217], mycosis fungoides [217], CD30-positive anaplastic large-cell lymphoma [217], lymphomatoid papulosis [218]	-
Extramammary Paget’s disease [219]	-
Vascular tumors (Kaposiform hemangioendothelioma with Kasabach-Merritt phenomenon and hypercalcemia [220,221]; blue rubber-bleb nevus [222])	-
Adnexal tumor (Pilomatricoma [223])	-

Hypervitaminosis A

Vitamin A toxicity, referred to as hypervitaminosis A, constitutes a severe and potentially lethal condition. Toxicity is dose-dependent; however, there is significant interindividual variability regarding the minimum intake necessary to induce toxicity [224]. The precise mechanism through which vitamin A may induce hypercalcemia remains unclear. It has been suggested that it exerts effects directly on the parathyroid, the bone, or both [225]. Vitamin A likely affects bone by either stimulating osteoclastic resorption, inhibiting osteoblastic synthesis, or both [226]. Chertow et al. have demonstrated that Vitamin A promotes PTH production in human and bovine parathyroid tissue [227].

*Hypervitaminosis D* 

Elevated circulating 25(OH)D plasma levels exceeding 160 ng/mL, accompanied by anorexia, nausea, and thirst, characterize hypervitaminosis D. Hypervitaminosis D results in elevated calcium enteric absorption and bone resorption, causing hypercalcemia. This condition subsequently leads to decreased PTH levels and a reduced glomerular filtration rate, ultimately disrupting calcium homeostasis. Calcinosis results from hypervitaminosis D, affecting organs such as the kidneys, bones, CNS, and cardiovascular system [228].

Connective Tissue Disorders

SLE

Hypercalcemia is relatively uncommon in SLE. Reports of hypercalcemia associated with SLE are limited. Stimulatory anti-PTH receptor antibodies have been proposed as a potential cause of hypercalcemia in patients with SLE [229]. A case report detailing hypercalcemia, widespread lymphadenopathy, and SLE in a patient has been reported. Immunohistology of two biopsied lymph nodes demonstrated elevated PTHrP expression without a trace of malignant transformation. Consequently, it was proposed that SLE patients might synthesize PTHrP from nonmalignant lymphoid tissue [230]. There are few reports of primary hyperparathyroidism in lupus patients. A case of primary hyperparathyroidism due to a parathyroid adenoma in a patient with SLE is documented, with hypercalcemia resolving following adenoma excision [231]. Secondary hyperparathyroidism in chronic renal failure associated with lupus nephritis [232].

Dermatomyositis

Life-threatening hypercalcemia in juvenile dermatomyositis has been associated with the regression of dystrophic calcifications [233,234]. Reports indicate a correlation between Dermatomyositis and malignancies accompanied by hypercalcemia [235,236]. Hypercalcemia is attributed to increased bone resorption resulting from the release of PTHrP and elevated extrarenal calcitriol synthesis by malignant lymphocytes [235,236].

Other Dermatological Conditions

A case report of Keratosis Ichthyosis Deafness (KID) syndrome has been documented in association with a lethal p.Ala88Val pathogenic variant in GJB2, accompanied by hypercalcemia of unknown etiology. Another case report details SAPHO (synovitis, acne, pustulosis, hyperostosis, osteitis) syndrome, presenting with hypercalcemia, with accelerated bone turnover as the potential explanation [237,238].

Drug-Induced Hypercalcemia

Drugs resulting in hypercalcemia are listed in Table 7.

**Table 7:** Dermatological drugs causing hypercalcemia. References: [239-241] FDA, Food and Drug Administration

	Medication	Mechanism of hypercalcemia
Vitamin D analogues	Calcipotriol	Hypercalcemia occurs when the dosage exceeds the FDA's maximum recommended weekly limit of 100 g for calcipotriol ointment, based on the standard formulation of 50 μg/g [239].
	Calcitriol	The effect on serum calcium is negligible, thus eliminating the risk of hypercalcemia [240].
	Tacalcitol	There is a single case report of hypercalcemia when topical tacalcitol was given with thiazide diuretic [241].

Phosphate

Phosphate is a vital electrolyte in the human body, comprising around 1% of total body weight. The typical serum phosphate concentration in adults varies between 2.5 and 4.5 mg/dL. Serum phosphate levels typically decline with age, with peak concentrations of 4.5 to 8.3 mg/dL observed in newborns, almost 50% greater than in adults, due to the heightened phosphate requirements for growth and development in infants and children. It is crucial for various metabolic processes, including muscular contraction, nerve conduction, skeletal mineralization, endochondral calcification, energy homeostasis, enzyme activity, and cell membrane integrity. Phosphate ions are essential in maintaining acid-base equilibrium by buffering hydrogen ions [242,243]. The phosphate equilibrium is regulated both directly and indirectly by 1α,25-dihydroxy vitamin D3, PTH, and the osteocyte-derived phosphatonin fibroblast growth factor 23 (FGF23) [243].

Hypophosphatemia

Hypophosphatemia in adults is defined by a blood phosphate level below 2.5 mg/dL. The etiologies of hypophosphatemia are categorized into three primary mechanisms: diminished phosphate intake or absorption, translocation of phosphate from the extracellular space to the intracellular space or bone, and phosphate loss through the renal route. The clinical signs of hypophosphatemia are contingent upon its severity and duration and are frequently nonspecific. Most of the patients are asymptomatic, with hypophosphatemia identified solely as an inadvertent discovery during laboratory evaluation. The manifestations of severe hypophosphatemia (serum levels of phosphate less than 1-1.5 mg/dL in adults) arise from the depletion of intracellular phosphate levels, reduced ATP availability, and diminished oxygen transport to tissues due to phosphate influencing the oxygen-carrying capacity of hemoglobin by modulating the production of 2,3-bisphosphoglycerate. Symptoms include fatigue, bone pain, muscle atrophy, hemolysis, compromised leukocyte function, myocardial dysfunction, cardiac arrhythmias, and acute respiratory failure. Treatment usually includes the use of oral supplements [242,243]. Dermatological diseases and drugs associated with hypophosphatemia are narrated below.

Cutaneous-Skeletal Hypophosphatemia Syndrome (CSHS)

CSHS is characterized by epidermal or melanocytic nevi, hypophosphatemic rickets, and high serum levels of the phosphatonin FGF23. Pathologically increased serum levels of FGF23, a hormone generated from bone that regulates phosphorus homeostasis, cause renal phosphate wasting (termed the *phosphaturic factor*) and impede renal 25(OH)-1-α-hydroxylase activity, resulting in diminished calcitriol synthesis. This leads to hyperphosphaturia, hypophosphatemia, and hence oncogenic hypophosphatemic osteomalacia (HO). Lim et al. have demonstrated high FGF23 levels and hypophosphatemia associated with multilineage somatic RAS mutations identified using exome sequencing of blood and afflicted skin tissue in four large epidermal nevi and one giant congenital melanocytic nevus [244]. There are reports of CSHS with linear verrucous nevi and hypophosphatemic rickets [245,246]. Reports indicate hypophosphatemic rickets in various epidermal syndromes, such as Schimmelpenning syndrome and Phacomatosis pigmentokeratotica [247,248].

Pseudoxanthoma Elasticum (PXE) and Generalized Arterial Calcification of Infancy (GACI)

PXE is an inherited, autosomal recessive, multisystemic disorder marked by ectopic mineralization and fragmentation of elastic fibers in soft connective tissues, including the skin, retina, and arterial blood vessels. GACI is an uncommon autosomal recessive disorder characterized by significant calcification of the internal elastic lamina of large- and medium-sized arteries, accompanied by intimal proliferation that results in arterial stenoses and heart failure within the initial months of life. Mutations in either ABCC6 (ATP binding cassette subfamily C member)or ENPP1 (ectonucleotide pyrophosphatase 1)can result in the severe phenotype of GACI, often culminating in mortality within the first year of life [249]. Mutations in ENPP1 can lead to typical pseudoxanthomatous skin lesions and angioid streaks of the retina in infants with GACI who have survived infancy; nevertheless, the later emergence of the *classic PXE* phenotype without GACI has only been seen in patients with mutations in ABCC6. Hypophosphatemic rickets is commonly reported in patients with ENPP1 mutations [250]. Certain patients may develop hypophosphatemic rickets accompanied by hyperphosphaturia, a condition linked to enhanced survival beyond infancy in individuals with GACI [250,251].

McCune-Albright Syndrome (MAS)

MAS is an uncommon genetic condition characterized by a triad of polyostotic fibrous dysplasia of bone, precocious puberty, and café-au-lait pigmentation of skin. MAS may be worsened by hypophosphatemia and diminished levels of 1,25(OH)2 D along with hypophosphatemia. Recent reports indicate that fibrous dysplasia (FD) tissue in patients with MAS expresses FGF23, recognized as a pathogenic phosphaturic factor [252,253].

Psoriasis Vulgaris

A singular case report of erythrodermic psoriasis associated with hypophosphatemia indicates a potential correlation between hypophosphatemia and the severity of psoriasis, as low serum phosphate levels were documented during two erythrodermic exacerbations, returning to normal during periods of remission. Correction of the hypophosphatemia resulted in an improvement of the erythroderma. However, methotrexate was necessary to achieve complete control of psoriasis. The explanation indicates that the levels of 1,25-dihydroxyvitamin D, which stimulates intestinal absorption of phosphate and calcium, have been demonstrated to decline inversely with the severity of psoriasis. Thus, this may represent an additional mechanism, alongside dermatogenic enteropathy, contributing to hypophosphatemia in erythrodermic psoriasis. Moreover, calcium and phosphate excretion occurs minimally in the epidermis, which may be augmented in erythrodermic psoriasis [254].

HO is a rare metabolic disorder marked by diminished serum phosphate levels, resulting in impaired mineralization of the bone matrix. HO generally comprises four prevalent types: X-linked dominant hypophosphatemia (XLH), autosomal dominant hypophosphatemic rickets (ADHR), tumor-induced osteomalacia (TIO), and random HO. A case report exists of sporadic HO associated with psoriasis [255].

TIO is a rare paraneoplastic condition resulting from tumors that secrete FGF23 [256]. Several studies have demonstrated an elevated risk of cancer in persons with psoriasis [257,258]. Two cases document TIO manifesting with hypophosphatemia in psoriasis [256,259]. A recently published paper by Okan et al. indicated that FGF23 levels are enhanced in psoriasis, and increased FGF23 is correlated with the severity of the disease [260]. In addition to its critical involvement in phosphate homeostasis, there is increasing evidence indicating that FGF23 also regulates immunological function. An increased concentration of FGF23 correlates with increased levels of inflammatory markers such as interleukin-6 (IL-6), C-reactive protein (CRP), fibrinogen, and tumor necrosis factor-alpha (TNFα), hence inducing inflammation [258,259]. Another theory is that FGF23 may indirectly provoke inflammation by reducing the concentration of 1,25-dihydroxyvitamin D. FGF23 inhibits the synthesis of 1,25-dihydroxyvitamin D and enhances its breakdown by suppressing renal 1alpha-hydroxylase and promoting 24-hydroxylase, respectively [260,261]. 1,25-dihydroxyvitamin D functions as an inhibitor of T-cell proliferation and other inflammatory mediators while also playing a role in suppressing keratinocyte proliferation and promoting its differentiation [258-262]. These findings indicate that elevated FGF23 levels may contribute to the aetiology of psoriasis. It was suggested that TIO might modify the clinical progression of psoriasis by secreting elevated levels of FGF23, leading to the worsening of psoriasis and hypophosphatemia. The preferred treatment for oncogenic osteomalacia is tumor excision. Complete excision of the tumor may reduce the clinical progression of the disease and improve biochemical markers, potentially resulting in a cure [263].

Von Recklinghausen Neurofibromatosis

There are few reports of HO associated with von Recklinghausen neurofibromatosis [264,265]. Osteomalacia in neurofibromatosis typically manifests later in adulthood, featuring renal phosphate loss accompanied by hypophosphatemia and numerous pseudofractures [266].

Abdel-Wanis and Kawahara [267] propose that a potential melatonin deficiency in neurofibromatosis-1 may contribute to the pathogenesis of hyperphosphaturia by diminishing sodium-phosphate cotransport, elevating cyclic adenosine monophosphate (cAMP) levels, enhancing the unopposed effect of dopamine on phosphate reabsorption, and raising glucocorticoid levels. Parathyroid hyperactivity due to osteomalacia may synergistically interact with dopamine, intensifying urinary phosphate loss. In contrast, the excessive production of corticosteroids will diminish nocturnal melatonin levels. Furthermore, hypercortisolism may impede melatonin secretion in the presence of hypophosphatemia, potentially leading to the advancement of bone deformities. Hypophosphatemic rickets/osteomalacia is likely attributable to elevated FGF23 release by cells originating from the nevus or neurofibromas [266]. Treatment for hypophosphatemic rickets typically involves a phosphate solution, with the addition of active vitamin D, specifically calcitriol, to prevent subsequent secondary hyperparathyroidism and improve intestinal phosphate absorption [268].

Acute Skin Failure

Hypophosphatemia is a prevalent consequence in patients with acute skin failure, exacerbating insulin resistance and affecting neurological status and diaphragmatic function [8].

Medication-Induced Hypophosphatemia

Medications that cause hypokalemia, hypomagnesemia, or metabolic acidosis may be linked to hypophosphatemia [269,270] and are enumerated in Table 8.

**Table 8:** Dermatological drugs induced hypophosphatemia. Reference: [271] HCO3, bicarbonate; CYP450, cytochrome P450; FGF23, fibroblast growth factor 23

Group	Drug	Mechanism
Antibiotics	Aminoglycosides	Drug-induced Fanconi syndrome (altered reabsorption of Phosphate, HCO3, glucose, amino acids, and uric acid from proximal tubules) resulting in elevated urine phosphate excretion.
	Linezolid	Intracellular phosphate shift resulting from mitochondrial reactivation after the cessation of linezolid, as linezolid directly disrupts mitochondrial protein synthesis and, subsequently, respiratory chain function.
	Rifampicin	Drug-induced Fanconi’s syndrome and the induction of CYP, resulting in enhanced degradation of calcidiol to inactive vitamin D metabolites.
	Isoniazid	Induction of CYP, which enhances the degradation of calcidiol to inactive vitamin D metabolites.
Antiviral	Acyclovir	Increased urinary phosphate excretion
	Cidofovir, Tenofovir	Drug-induced Fanconi syndrome
	Lamivudine, Entecavir, Protease inhibitors (Darunavir, Lopinavir, Atazanavir)	
Tyrosine inhibitors	Imatinib mesylate, sorafenib, imatinib, sunitinib ibrutinib, dasatinib, dabrafenib, nilotinib, and ceritinib	Tubular dysfunction and Fanconi's syndrome, leading to inappropriate phosphaturia, or secondary hyperparathyroidism due to hypocalcemia linked to tyrosine kinase inhibitors
Phosphodiestersae inhibitors	Apremilast	Drug-induced Fanconi’s syndrome
Bisphosphanates		Increased urinary phosphate excretion
Platinum-based chemotherapy	Cisplatin	Tubular damage and hypomagnesemia-induced phosphaturia, resulting in hypophosphatemia
Vitamin	Niacin	Decreased intestinal absorption of phosphate
Immunosuppressants	Corticosteroids	Hypercortisolism in Cushing's syndrome can cause hypophosphatemia by elevating urine phosphate excretion or by impairing intestinal phosphate absorption. This process may be facilitated by FGF23 [271].

Hyperphosphatemia 

Hyperphosphatemia is characterized by abnormally elevated blood phosphate levels, exceeding 4.5 mg/dL, due to several underlying causes. 

(1) Acute phosphate overload resulting from the consumption of phosphate-containing laxatives, vitamin D toxicity, tumor lysis syndrome, and rhabdomyolysis.

(2) Reduced phosphate excretion was observed in renal failure, hypoparathyroidism, and pseudohypoparathyroidism.

(3) Transcellular transfer from intracellular to extracellular compartment: diabetic ketoacidosis and lactic acidosis.

The majority of patients are asymptomatic or display symptoms related to the underlying etiology of hyperphosphatemia. Acute hyperphosphatemia may manifest with hypocalcemia symptoms due to the binding of excess phosphate ions to calcium, resulting in decreased serum calcium levels, which manifests as this condition leads to the development of symptoms such as muscle cramps, perioral numbness, tingling, and tetany. Hyperphosphatemia in chronic kidney disease patients can elevate the risk of arterial calcification, hence increasing the likelihood of cardiovascular events [242].

Calciphylaxis

Calciphylaxis, often known as calcific uremic arteriolopathy, predominantly affects individuals with end-stage renal disease (ESRD). Patients often have concomitant hyperphosphatemia, an elevated calcium-phosphate product, and hyperparathyroidism [272]. Metastatic calcinosis cutis is often associated with hypercalcemia, hyperphosphatemia, or hyperparathyroidism [273].

Hypervitaminosis D

Hypervitaminosis D increases intestinal phosphorus uptake and decreases PTH levels, both leading to hyperphosphatemia [274].

Drug-Induced Hyperphosphatemia

Bisphosphonates cause mild to moderate hyperphosphatemia by increasing phosphorus reabsorption [275].

Magnesium

Mg is the second-most abundant intracellular divalent cation in the body. The typical reference range is 0.7-1 mmol/L (1.5-2 mEq/L or 1.7-2.4 mg/dL) [276]. Mg plays a pivotal role in nucleic acid and protein synthesis, neuromuscular conduction, cardiac contractility, energy metabolism, and immune system function [277]. Mg homeostasis is regulated by three distinct organ systems: the intestines, bones, and kidneys. The PTH regulates Mg absorption. Additional dietary factors are also implicated. These comprise vitamin D, oxalate, phosphate, proteins, potassium, and zinc [278].

Hypomagnesemia

Hypomagnesemia impedes the Mg-dependent production of cAMP induced by adenyl cyclase, leading to the diminished release of PTH and subsequently lowering calcium levels, as PTH maintains calcium homeostasis. Hypomagnesemia is generally characterized by blood Mg levels below 0.7 mmol/L, with or without total body depletion, and does not result in clinically relevant signs and symptoms until serum levels decline below 0.5 mmol/L [279].

Clinical manifestations include:

Neuromuscular: hyperexcitability, weakness, apathy, delirium, and coma

Cardiovascular: QRS widening and peak T-waves with moderate Mg depletion, widening of the PR interval, diminution of T waves, and atrial and ventricular arrhythmias with severe depletion

Other coexisting electrolyte abnormalities include hypokalemia, hypocalcemia, metabolic alkalosis, and hypoparathyroidism [280]. Dermatological conditions and medication linked to hypomagnesemia are narrated next.

Vitiligo Vulgaris

Hypomagnesemia has been demonstrated to exaggerate oxidative stress and inflammation. A study by Namazi et al. observed a positive correlation between serum Mg levels and the Vitiligo Area Severity Index (VASI) score and the total body surface area (TBSA) affected by the disease [280].

Mycosis Fungoides (MF)

Hypomagnesemia has been suggested as a potential cause of immunological dysregulation disorders. A case series by Morgan et al. provides conclusive evidence indicating a correlation between reduced serum Mg levels and the advancing stages of Mycosis fungoides, highlighting its significance in the pathophysiology and progression of MF and Sézary syndrome [281].

Toxic Shock Syndrome (TSS)

In vitro investigations have indicated that peak Staphylococcus aureus toxin production occurs in a low-Mg environment. A study by Kass [282] proposed that Mg deficiency may contribute to the pathophysiology of TSS. TSS will manifest in individuals harboring staphylococci that generate toxic shock syndrome toxin-1 (TSST-1) and who are deficient in the associated antibody. 

Mg deficit may result from the influence of certain polyacrylate fibers previously used in tampons combined with Mg+ and maybe other mechanisms that create a low Mg environment for staphylococci, facilitating toxin production. During relatively high menstrual flow, sufficient Mg exists in the blood and cellular debris to fully saturate the binding capacity of the fibers while still maintaining an adequate concentration of unbound ions to ensure that staphylococci have a sufficiently elevated level of Mg in the environment, thereby minimizing toxin production. Conversely, when menstrual flow is reduced, the quantity of Mg extracted by specific fibers may be sufficient to create a low-Mg environment, resulting in a significant rise in the generation of TSST-1 and other toxins that could be influenced by Mg concentration. The proposed hypothesis elucidates various characteristics of TSS. The peak attack rate of TSS occurs on the fourth day of menstruation; this is likely a period when menstrual flow is comparatively minimal. However, due to the irregularity of menstrual flow, reduced amounts may also manifest less frequently on other days within the menstrual cycle. Likewise, numerous studies have suggested that sexually active women appear to be less prone to getting TSS compared to sexually inactive women. Given that human semen contains approximately tenfold the concentration of Mg ions compared to blood, this excess Mg may likely persist in the genital tract sufficiently to confer some protection against Mg insufficiency and the resultant release of excess toxins. The material used in tampons has been revolutionized with cotton or rayon or a blend of both [282].

AD

The role of Mg in AD remains unexplored; nonetheless, exposure to Mg-rich water has demonstrated positive effects on the skin barrier in individuals with dry atopic skin [283]. Mg plays a role in the synthesis of ceramides and the regulation of epidermal proliferation and differentiation. Furthermore, children with AD exhibited lower serum Mg levels [284].

Rattanatayarom et al. reported a hypomagnesemia frequency of 0% in controls, 10% in Crohn’s disease (CD), and 16.7% in AD, based on a sample of 30 patients per group. They also proposed that atopic patients be routinely tested for low Mg levels so that targeted Mg supplements may be administered [285].

*Alopecia* 

A study by Tataru and Nicoara found a correlation between low serum Mg levels and idiopathic diffuse alopecia in young women. It was revealed that young women with idiopathic diffuse alopecia exhibited a significantly higher percentage of hypomagnesemia status compared to the control group (46.1% vs. 8.3%). Women with diffuse alopecia of distinct etiology exhibited a significantly higher hypomagnesemia status compared to the control group. The supplementation therapy with Mg, along with vitamin B6, was effective in improving hair loss in young women with idiopathic diffuse alopecia [286].

Porphyria

Severe hypomagnesemia can arise during acute porphyria episodes and is a recognized precipitant of generalized seizures; it must be rapidly rectified with intravenous Mg sulfate [287].

Plasmapheresis

Plasmapheresis is employed for several autoimmune disorders, including Goodpasture syndrome, lupus erythematosus, pemphigus vulgaris, and bullous pemphigoid [288]. Ionized hypomagnesemia may be noted during plasmapheresis and was attributed to the chelation of Mg2+ by the citrate present in fresh frozen plasma and anticoagulant citrate dextrose. Hemodilution may induce ionized hypomagnesemia [289].

*Helix Syndrome* 

Helix syndrome is a rare disorder characterized by hypermagnesemia and hypomagnesuria. A diagnosis is established when dyselectrolytemia and hypermagnesemia are present alongside somatic manifestations of hypohidrosis, lacrimal gland dysfunction, ichthyosis, and xerostomia. This condition is due to a mutation of CLDN10B, which encodes Claudin-10b, a tight junction (TJ) membrane-spanning protein expressed in the skin, salivary glands, and kidneys. CLDN10 mutations cause dysfunction in TJs in several tissues and, subsequently, abnormalities in epidermal integrity, ectodermal gland homeostasis, and renal ion transport [290].

Hypochloric Acid-Induced Injury/Burn

There are reports of hypomagnesemia and hypocalcemia accompanied by hypokalemia following a chemical injury with exposure to hydrofluoric acid induced by binding with fluoride anion [291,166].

Salicylate Toxicity

Salicylate toxicity can also present with hypomagnesemia along with other metabolic disturbances such as hypokalemia and hypocalcemia [292].

Drug-Induced Hypomagnesemia

Drugs causing hypomagnesemia are detailed in Table 9.

**Table 9:** Drugs causing hypomagnesemia. References: [293-295]. TAL, thick ascending limb; CNIs, calcineurin inhibitors; TRPM, transient receptor potential cation channel subfamily M member; EGF, epidermal growth factor; RANKL, receptor activator of nuclear factor-kappa B ligand; EGFR, epidermal growth factor receptor; mTOR, mammalian target of rapamycin; DCT, distal convoluted tubule; PPI, proton pump inhibitors

Class	Drug	Cause	Additional points
Antibiotics	Aminoglycosides (gentamicin, amikacin, tobramycin, and capreomycin)	Activation of the calcium-sensing receptor situated on the basolateral membrane of the thick ascending limb (TAL) results in the suppression of tubular transport in this segment and paracellular transport of magnesium, leading to increased renal excretion and subsequent hyperaldosteronism.	Hypomagnesemia may develop and persist in relation to both duration and dose, even after the discontinuation of aminoglycoside therapy.
Antifungal	Amphotericin B	Tubular membrane disruption with enhanced permeability and tubular injury/necrosis [293].	It is more common with the deoxycholate than with the lipid formulation.
	Posaconazole and Isavuconazole		
Antiviral	Foscarnet	Increased renal magnesium loss, a potent chelation of divalent ions, leads to ionized hypomagnesemia.	
Antiprotozoal	Pentamidine	Increased renal magnesium loss may lead to acute pancreatitis, contributing to hypomagnesemia due to the saponification of magnesium in necrotic fat.	
Calcineurin inhibitors (CNIs)	Cyclosporine, Tacrolimus	CNIs induce excessive renal magnesium loss, presumably by decreasing the expression of TRPM6; additionally, a translocation of magnesium into cells may also play a role.	CNI-induced hypomagnesemia is typically mild; however, severe neurological symptoms, such as altered mental status, seizures, and focal neurological deficits, have been documented. Hypomagnesemia is suggested to contribute to the nephrotoxic effects and increased blood pressure linked to calcineurin inhibitors (CNIs).
Antineoplastic agents	Platinum‐containing drugs (cisplatin, carboplatin, and oxaliplatin)	Downregulation of the TRPM6/EGF pathway	
Anti-EGFR inhibitors	Cetuximab, Panitumumab, Zalutumumab	Inactivation of EGFR results in TRPM6 downregulation, resulting in renal Magnesium depletion.	The incidence of hypomagnesemia and hypokalemia was higher with panitumumab compared to cetuximab or bevacizumab, whereas zalutumumab has been linked to lower rates of hypomagnesemia (4%) and hypokalemia (6%).
mTOR Inhibitors	Sirolimus	Decrease in TRPM6 expression in the DCT as a result of diminished TRPM6 mRNA stability.	
PPI	Omeprazole, Esomeprazole, Pantoprazole, and Rabeprazole	Diminished active Magnesium transport in the colon, primarily mediated by the ion channels TRPM6 and TRPM7, serves as the principal explanation for proton pump inhibitor-related hypomagnesemia.	
Drugs used in osteoporosis	Bisphosphonates and Denosumab (RANKL ligand inhibitor)	Hypomagnesemia is due to their binding to Mg cations [294,295].	

## Conclusions

An in-depth investigation of electrolyte imbalances in dermatology and its ramifications is necessary to address the infrequent, although potential, adverse outcomes. Both conventional medications, including antibiotics, antivirals, and antifungals, as well as unusual molecular-focused medicines, have shown electrolyte abnormalities. The medicine's mechanism of action, its targeted pathways, and the selection of suitable individuals for the therapy are crucial. Understanding infectious etiologies and their pathophysiology is essential for comprehending the changes in electrolytes. Alongside the common clinical conditions, autoimmune illnesses and immunodeficiency syndromes also induce metabolic derangements characterized by serum electrolyte disturbances. Electrolyte abnormalities are rare but may arise in dermatological diseases. Dermatologists must remain vigilant to initiate treatment promptly and avert problems.
